# Genome-wide analysis of the Hsf family in soybean and functional identification of *GmHsf-34* involvement in drought and heat stresses

**DOI:** 10.1186/1471-2164-15-1009

**Published:** 2014-11-21

**Authors:** Pan-Song Li, Tai-Fei Yu, Guan-Hua He, Ming Chen, Yong-Bin Zhou, Shou-Cheng Chai, Zhao-Shi Xu, You-Zhi Ma

**Affiliations:** College of Agronomy, Northwest A & F University, Yangling, 712100 China; Chinese Academy of Agricultural Sciences (CAAS)/National Key Facility for Crop Gene Resources and Genetic Improvement, Key Laboratory of Biology and Genetic Improvement of Triticeae Crops, Ministry of Agriculture, Institute of Crop Science, Beijing, 100081 China

**Keywords:** Hsfs, Genome-wide identification, Expression pattern, Subcellular localization, Functional identification, Soybean

## Abstract

**Background:**

High temperature affects organism growth and metabolic activity. Heat shock transcription factors (Hsfs) are key regulators in heat shock response in eukaryotes and prokaryotes. Under high temperature conditions, Hsfs activate heat shock proteins (Hsps) by combining with heat stress elements (HSEs) in their promoters, leading to defense of heat stress. Since the first plant Hsf gene was identified in tomato, several plant Hsf family genes have been thoroughly characterized. Although soybean (*Glycine max*), an important oilseed crops, genome sequences have been available, the Hsf family genes in soybean have not been characterized accurately.

**Result:**

We analyzed the Hsf genetic structures and protein function domains using the GSDS, Pfam, SMART, PredictNLS, and NetNES online tools. The genome scanning of dicots (soybean and *Arabidopsis*) and monocots (rice and maize) revealed that the whole-genome replication occurred twice in soybean evolution. The plant Hsfs were classified into 3 classes and 16 subclasses according to protein structure domains. The A8 and B3 subclasses existed only in dicots and the A9 and C2 occurred only in monocots. Thirty eight soybean Hsfs were systematically identified and grouped into 3 classes and 12 subclasses, and located on 15 soybean chromosomes. The promoter regions of the soybean Hsfs contained *cis*-elements that likely participate in drought, low temperature, and ABA stress responses. There were large differences among Hsfs based on transcriptional levels under the stress conditions. The transcriptional levels of the A1 and A2 subclass genes were extraordinarily high. In addition, differences in the expression levels occurred for each gene in the different organs and at the different developmental stages. Several genes were chosen to determine their subcellular localizations and functions. The subcellular localization results revealed that GmHsf-04, GmHsf-33, and GmHsf-34 were located in the nucleus. Overexpression of the *GmHsf-34* gene improved the tolerances to drought and heat stresses in *Arabidopsis* plants.

**Conclusions:**

This present investigation of the quantity, structural features, expression characteristics, subcellular localizations, and functional roles provides a scientific basis for further research on soybean Hsf functions.

**Electronic supplementary material:**

The online version of this article (doi:10.1186/1471-2164-15-1009) contains supplementary material, which is available to authorized users.

## Background

Heat stress, defined as a rise in the temperature of 10-15°C above the ambient [1], beyond a given threshold level for a period of time, is an agricultural problem in many areas all over the world, affecting plant growth and development and often leading to reductions in yield. All organisms, including eukaryotes and prokaryotes, share a common heat shock response mechanism, which involves a number of reactions, including new protein synthesis, folding, intracellular targeting, specific biological functions, and protein degradations. Among these proteins, Hsps acting as molecular chaperones are essential for the maintenance and/or restoration of protein homeostasis [2–8]. The Hsp expression is regulated by the multiple mechanisms. The central regulators are Hsfs. Under high temperature conditions, Hsfs activate Hsps by combining with HSEs in their promoters, leading to the defense of the heat stress and even recovering from its effects.

A typical Hsf protein contains a modular structure with an N-terminal DNA-binding domain (DBD), an adjacent oligomerization domain (OD) composed of heptad repeats of hydrophobic amino acid residues (HR-A/B), a nuclear localization signal (NLS) region essential for nuclear uptake of the protein, a nuclear export signal (NES) region, and an activator motif (AHA) [9]. *Arabidopsis* Hsfs were classified into A, B, and C classes according to the differences in their HR-A/B regions. Due to the insertion of 21 (class A) or 7 (class C) amino acid residues between the A and B parts of the HR-A/B regions, the class A and class C Hsfs have longer HR-A/B regions than class B, which is distinguished from class A and C by the presence of a heptad repeat pattern instead of an insertion. Unlike class B and class C, the class A members contain a C-terminal AHA motif relevant to their own activator function, and a hydrophobic, frequently leucine-rich NES required for the receptor-mediated nuclear export in complex with the NES receptor [10].

Under normal circumstances, the inactive state of a monomeric Hsf is maintained by the interaction with the molecular chaperones, such as Hsp70 and Hsp90. In response to heat stress, Hsfs released from the chaperone complex are converted from a transcriptional inactive monomer to an active trimmer through combination of their ODs. As sequence-specific trimeric DNA binding proteins, the active Hsfs are capable of recognizing and combining HSEs in the Hsf-inducible gene promoters [11]. HSEs are formed of repetitive palindromic binding motifs of the 5’-AGAAnnTTCT-3’ sequence upstream of the TATA box in the Hsf-inducible genes [12–15].

Since the first plant Hsf gene was identified in tomato [16], the Hsf family genes have been thoroughly characterized, and 21, 25, 25, and 27 Hsf genes were found in *Arabidopsis*, rice, maize, and tomato, respectively [9, 17–19]. In the present study, we scanned for and integrated all the nonredundant sets of the soybean Hsf genes, determined their chromosomal locations, predicted their protein structures by available software and network stations, analyzed the expression levels of the soybean Hsf genes by qRT-PCR and identified the function of *GmHsf-34* in the tolerance to drought and heat stresses. This study provides a version on the structures and evolutionary history of the soybean Hsfs, and a candidate gene to the crop molecular breeding.

## Results

### Identification, phylogenetic, and evolutionary analyses

The amino acid sequences of Hsf-type DBD domains (Pfam: PF00447) were submitted into JGI Glyma1.0 annotation for BLASTP searches. Fifty-eight putative soybean Hsf sequences were acquired. After surveyed using the Pfam database and SMART online tool, 4 soybean Hsf sequences were rejected due to the absence of typical Hsf DBD domains, and 16 were abandoned due to the absence of coiled-coil structures. Consequently, 38 nonredundant soybean Hsfs were identified (Table 1). The polypeptide lengths of soybean Hsfs varied widely, ranging from 213 to 510. Isoelectric points of the proteins were diverse (Table 1).

**Table 1:** **Protein information of soybean Hsfs, including sequenced ID, protein sequence length, molecular weight (MW), isoelectric point (pI), and chromosome locations**

Number	Gene name	Gene ID number	Amino acid residues	MW (Da)	pI	Chromosome
1	*GmHsf-01*	Glyma01g39260	282	31188.2	9.23	1
2	*GmHsf-02*	Glyma01g01990	461	50834.4	5.20	1
3	*GmHsf-03*	Glyma01g42640	338	36916.2	4.92	1
4	*GmHsf-04*	Glyma01g44330	464	51837.6	4.69	1
5	*GmHsf-05*	Glyma01g34490	209	24224.3	7.47	1
6	*GmHsf-06*	Glyma03g29190	231	26709.6	8.89	3
7	*GmHsf-07*	Glyma03g34900	423	48259.8	5.54	3
8	*GmHsf-08*	Glyma04g05500	372	41915.4	4.85	4
9	*GmHsf-09*	Glyma05g28460	479	54019.5	5.37	5
10	*GmHsf-10*	Glyma05g34450	358	41011.2	5.07	5
11	*GmHsf-11*	Glyma05g29470	382	43798.1	4.78	5
12	*GmHsf-12*	Glyma05g20460	322	35003.5	6.26	5
13	*GmHsf-13*	Glyma08g12630	402	45945.1	4.85	8
14	*GmHsf-14*	Glyma08g05220	364	41709.8	4.93	8
15	*GmHsf-15*	Glyma08g11460	477	53887.4	5.82	8
16	*GmHsf-16*	Glyma09g32300	320	35969.4	6.74	9
17	*GmHsf-17*	Glyma09g33920	500	55523.0	4.68	9
18	*GmHsf-18*	Glyma09g26510	324	35578.3	5.79	9
19	*GmHsf-19*	Glyma10g38240	289	32541.0	7.54	10
20	*GmHsf-20*	Glyma10g07620	435	48901.5	5.89	10
21	*GmHsf-21*	Glyma10g03530	341	39735.3	5.86	10
22	*GmHsf-22*	Glyma10g00560	324	37691.8	4.35	10
23	*GmHsf-23*	Glyma10g38930	448	52050.0	6.34	10
24	*GmHsf-24*	Glyma11g01190	464	52011.0	5.22	11
25	*GmHsf-25*	Glyma11g06010	285	31579.6	9.45	11
26	*GmHsf-26*	Glyma11g02800	355	38645.8	4.71	11
27	*GmHsf-27*	Glyma13g24860	213	24793.9	7.85	13
28	*GmHsf-28*	Glyma13g21490	428	48226.5	4.93	13
29	*GmHsf-29*	Glyma13g29760	392	44926.1	4.62	13
30	*GmHsf-30*	Glyma14g11030	362	41044.5	4.52	14
31	*GmHsf-31*	Glyma15g09280	392	44871.2	4.85	15
32	*GmHsf-32*	Glyma16g32070	348	37752.1	6.52	16
33	*GmHsf-33*	Glyma16g13400	510	56338.6	4.68	16
34	*GmHsf-34*	Glyma17g34540	335	38020.3	4.97	17
35	*GmHsf-35*	Glyma17g20070	282	30976.9	6.30	17
36	*GmHsf-36*	Glyma19g31940	233	26782.8	9.92	19
37	*GmHsf-37*	Glyma20g28870	341	39444.9	4.93	20
38	*GmHsf-38*	Glyma20g29610	300	33289.6	8.71	20

**Figure 1:**
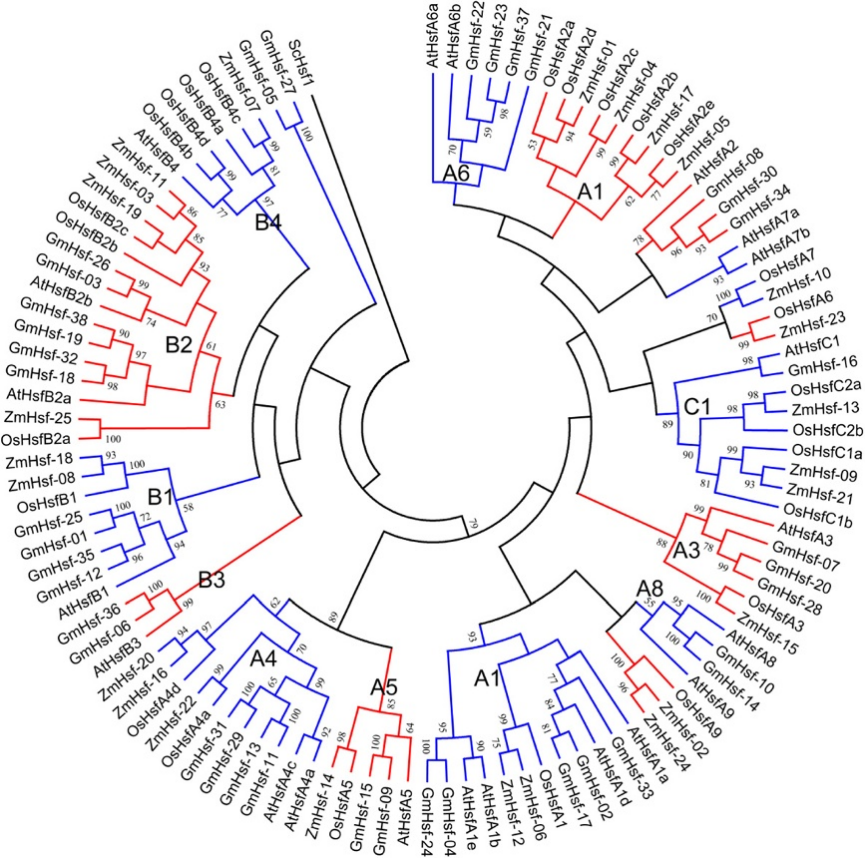
**Phylogenetic relationship of the Hsfs involving with Gm (** ***Glycine max*** **), At (** ***Arabidopsis thaliana*** **) Os (** ***Oryza sativa*** **), and Zm (** ***Zea mays*** **).** The phylogenetic tree is produced by MEGA 5.0 software based on the comparison of amino acid sequences of the DNA binding domain, the HR-A/B region and the linker between these two regions. ScHsf1 was used as the out group. The neighbor-joining method was used and the bootstrap values were set at 1000. The frequency values (%) higher than 50 were showed nearby the branch lines. GmHsfs were divided into 3 classes and 12 subclasses (A1, A2, A3, A4, A5, A6, A8, B1, B2, B3, B4 and C1) and separated by red and blue branches. In this analysis, AtHsfA9 was classified into A8 subclasses.

To determine the phylogenetic relationships among soybean Hsfs, a phylogenetic analysis of 38 soybean Hsfs, 25 maize Hsfs, 25 rice Hsfs, and 21 *Arabidopsis* Hsfs was performed by generating a neighbor-joining phylogenetic tree (Figure 1). According to differences in the amino acid sequences of DBD, the HR-A/B region, and the linker between them, the A, B, and C Hsf classes formed three clusters. Class A was divided into 10 sub-clusters, designated A1, A2, A3, A4, A5, A6, A7, A8, A9, and A10. Class B was divided into sub-clusters B1, B2, B3, and B4, and the class C contains sub-clusters C1 and C2. Soybean Hsfs were further divided into 12 sub-clusters according to their phylogenetic relationship, defined as A1, A2, A3, A4, A5, A6, A8, B1, B2, B3, B4, and C1 (Figure 1). As a dicot, soybean was more similar to *Arabidopsis* than to the monocots rice and maize. AtHsf-09 and AtHsf-10 were the only two members of subclass A7. The A8 and B3 subclasses were present only in the dicots, and A9 and C2 existed only in the monocots. Interestingly, soybean subclass B4 had higher similarity to *Arabidopsis* B4 than to the rice or maize B4 subclasses, and soybean subclass A6 Hsfs showed higher similarity to A4 rather than to *Arabidopsis* subclass A6.

### Physical locations of soybean Hsfs

**Figure 2:**
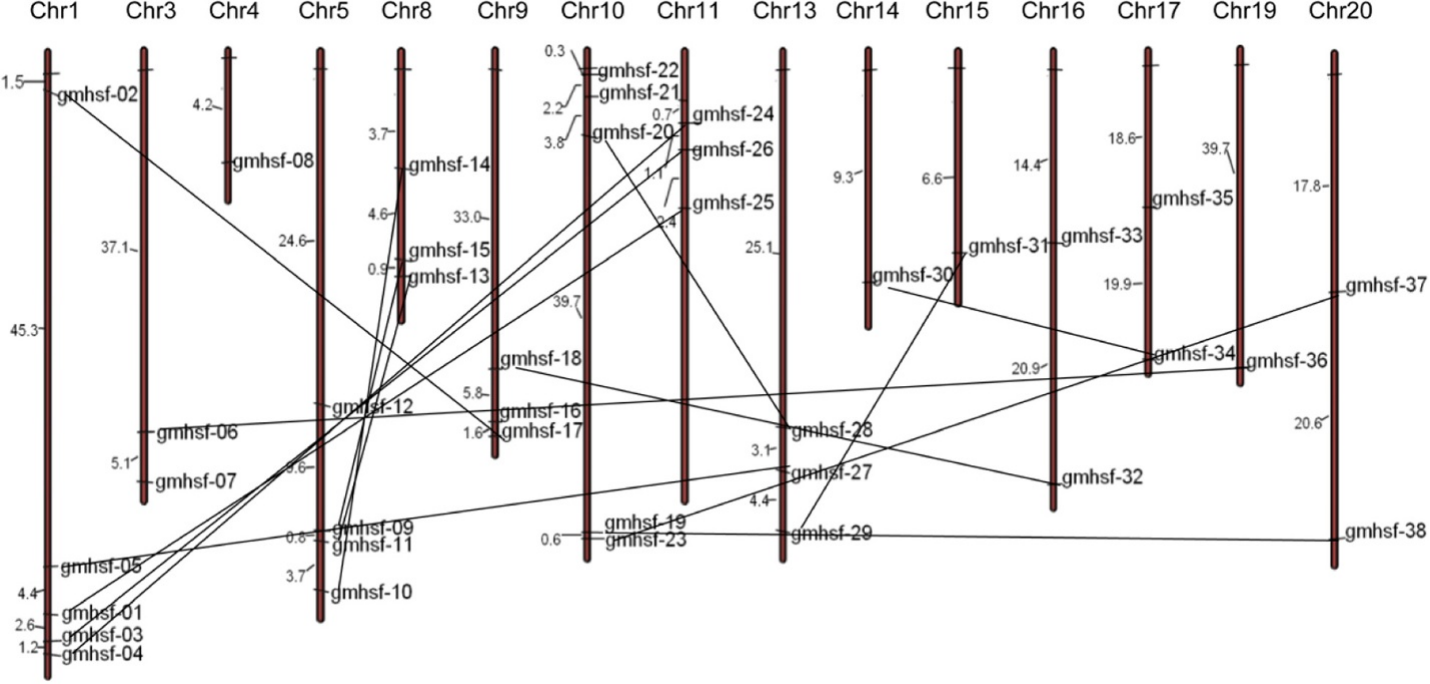
**Distribution and duplications of soybean Hsf genes in soybean genome.** The brown bars represent the chromosomes and the chromosome numbers are showed on the top of the bars. But the length of the bar has no relationship with the size of the chromosomes. GmHsf genes distribute on the 15 chromosomes. The numbers on the left side of the chromosomes show the distances between the neighboring genes and unit of the distance is megabase (Mb). The paralogous genes are identified and connected by lines.

According to the soybean genome database, 38 soybean Hsf genes were distributed among 15 chromosomes, with the exception of chromosome 2, 6, 7, 12, and 18 (Figure 2). The number of soybean Hsf genes in each chromosome differed considerably. For example, chromosome 1 and 10 carried 5 soybean Hsf genes, whereas only one was present in chromosome 4, 14, and 19 respectively. Using soybean genome repeat informations, 15 paralogous genes were identified (Figure 2).

### Gene structures and *cis*-acting elements

**Figure 3:**
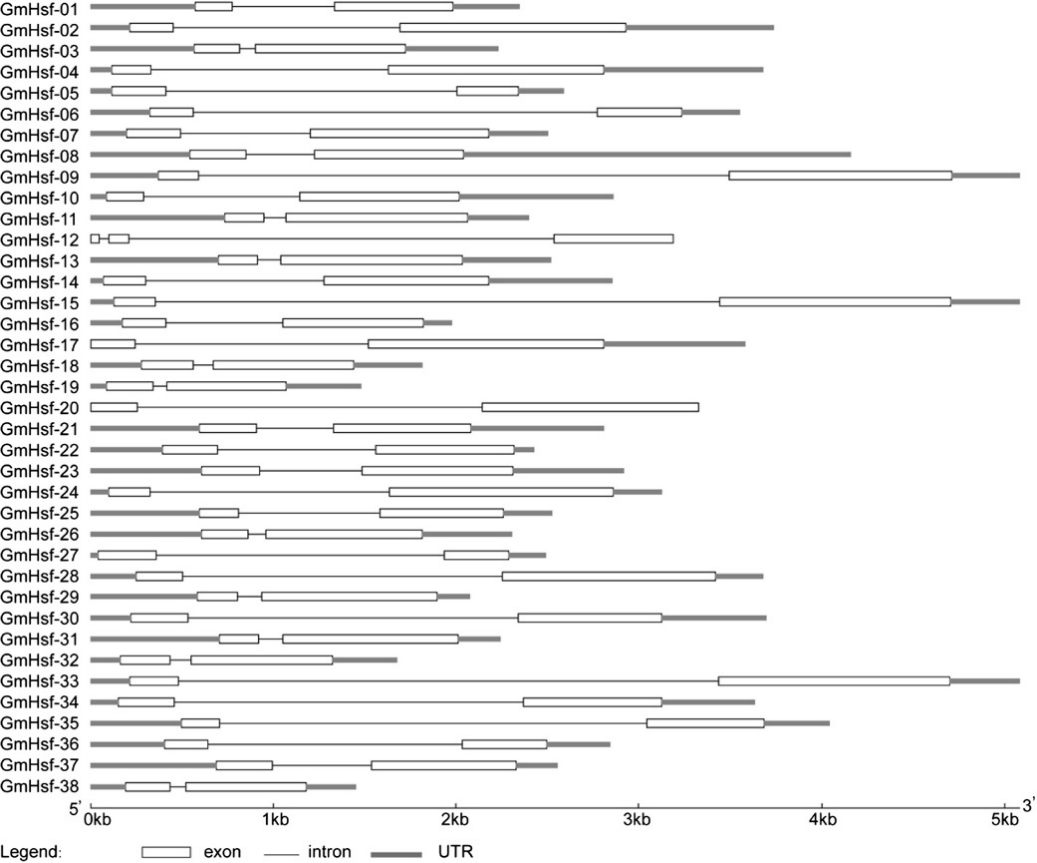
**Intron-exon structures of soybean Hsf genes.** The Intron-exon structures were produced by the GSDS online tool. The exons, introns and untranslated regions (UTRs) were indicated by the white boxes, black lines and gray lines respectively.

Gene structure analysis revealed the existence of introns in the soybean Hsf genes. Four introns were found in *GmHsf-20*, 3 in *GmHsf-23*, and 2 in *GmHsf-02*, *GmHsf-12*, *GmHsf-17*, and *GmHsf-18* respectively (Figure 3). *Cis*-element analysis demonstrated that every soybean Hsf member carried one or more MYB and MYC elements in their promoters. In addition, 52.6% of the members contained an ABA-responsive element (ABRE), 31.6% contained a dehydration-responsive element (DRE), and 42.1% contained a low-temperature responsive element (LTRE) (Table 2). It was reported that the above-mentioned 5 elements play different significant roles in stress responses in plants. For example, MYB is involved in stress-induced drought, low temperature, salt, ABA, and GA responses [20]. ABRE responds to drought and ABA *via* combination with ABRE binding proteins (AREB) [21]. DRE combining with DRE binding proteins (DREB) participate in drought, salt, low temperature, and ABA responses [22]. LTRE contributes primarily to low temperature response and regulation [23]. Analyses of *cis*-elements in the promoters suggest that Hsfs are significantly related to stress response.

**Table 2:** **Distribution of ABRE, DRE, LTRE, MYB, and MYC** ***cis*** **-acting elements in soybean Hsf promoters**

Gene	ABRE	DRE	LTRE	MYB	MYC
*GmHsf-01*	0	0	0	7	6
*GmHsf-02*	1	2	0	6	2
*GmHsf-03*	0	0	0	2	2
*GmHsf-04*	0	3	0	11	8
*GmHsf-05*	4	0	0	21	30
*GmHsf-06*	3	0	0	28	22
*GmHsf-07*	3	0	0	28	22
*GmHsf-08*	4	1	0	22	28
*GmHsf-09*	0	0	0	25	12
*GmHsf-10*	0	2	2	31	50
*GmHsf-11*	0	0	0	7	10
*GmHsf-12*	0	2	1	5	4
*GmHsf-13*	1	0	0	7	6
*GmHsf-14*	0	0	0	26	26
*GmHsf-15*	0	0	4	19	12
*GmHsf-16*	3	0	0	6	6
*GmHsf-17*	4	3	0	33	38
*GmHsf-18*	3	0	2	31	8
*GmHsf-19*	6	1	2	15	10
*GmHsf-20*	0	0	0	5	4
*GmHsf-21*	1	0	0	39	6
*GmHsf-22*	1	0	1	14	12
*GmHsf-23*	1	0	0	7	10
*GmHsf-24*	0	3	1	11	4
*GmHsf-25*	0	0	0	9	6
*GmHsf-26*	0	1	3	22	2
*GmHsf-27*	0	0	0	7	6
*GmHsf-28*	0	0	0	13	2
*GmHsf-29*	2	0	1	1	2
*GmHsf-30*	0	0	0	23	22
*GmHsf-31*	0	0	1	12	18
*GmHsf-32*	9	1	3	47	8
*GmHsf-33*	3	0	1	13	12
*GmHsf-34*	6	0	1	23	22
*GmHsf-35*	4	2	1	19	10
*GmHsf-36*	2	2	1	14	24
*GmHsf-37*	0	0	0	12	10
*GmHsf-38*	9	0	1	16	8

### Conserved domains and motifs of soybean Hsfs

**Figure 4:**
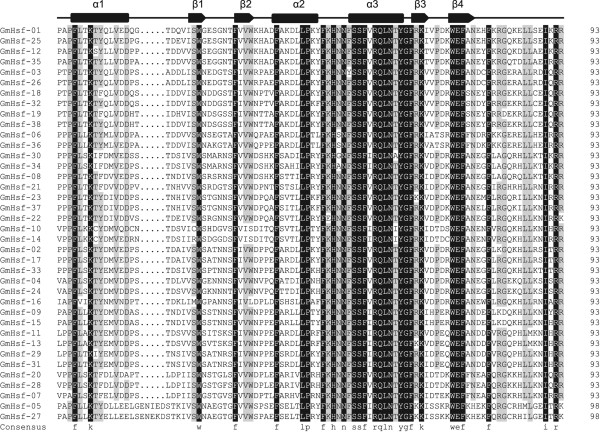
**Multiple sequence alignment of DNA binding domains of soybean Hsfs.** DBD domain sequences of GmHsfs identified by Pfam database were aligned by Clustal X 2.0 software and edited by DNAMAN software. The black and gray backgrounds indicate entire conservative residues and 75% conservative residues respectively. The helix-turn-helix motifs of DBD (α1-β1-β2-α2-α3-β3-β4) are shown at the top. Cylindrical tubes represent α-helices and block arrows represent β-sheets.

**Figure 5:**
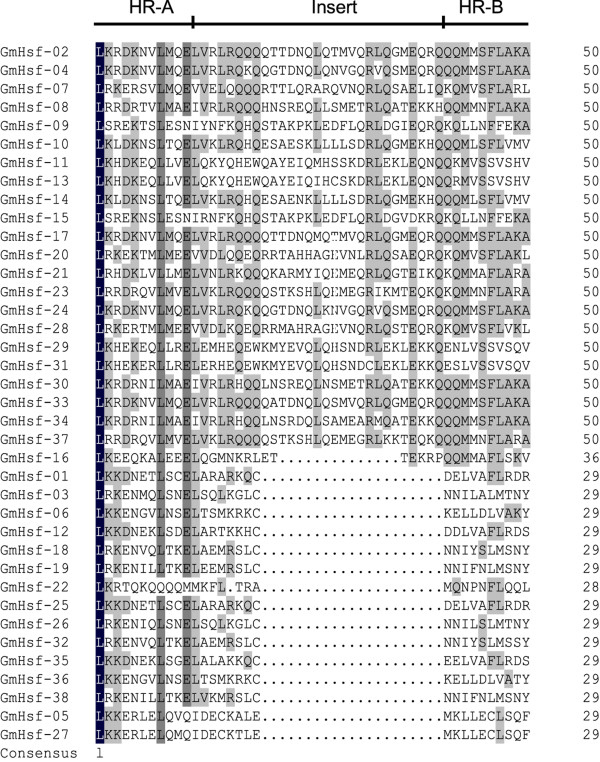
**Multiple sequence alignment of the HR-A/B regions (OD) of soybean Hsfs.** The HR-A/B region sequences identified by SMART online tool were aligned by Clustal X 2.0 software and edited by DNAMAN software. The black and gray backgrounds indicate the 100% conservative residues and 50-75% conservative residues respectively. The three line segments at the top divide HR-A core, insert and HR-B regions orderly.

The modular structure of the Hsf family in plants has been described thoroughly in several model plants [9, 18, 24]. A typical soybean Hsf protein contains 5 conserved domains. There is a gradation of DBD, OD, NLS, NES, and AHA domains from N-terminal to C-terminal (Table 3). The DBD domain, the most conserved section, composed of approximately 100 amino acids, contains 3 α-helices and a four-stranded antiparallel β-sheet (α1-β1-β2-α2-α3-β3-β4) (Figure 4). This helix-turn-helix motif (H2-T-H3) specifically combines to HSEs in the promoters of heat-stress-induced genes. HR-A/B adjacent to the DBD domain in the C-terminal is characterized by a coiled-coil structure (coil-coil structure). According to the distinction between the HR-A and HR-B motifs, Hsfs were artificially divided into A, B, and C classes. Because of the insertion of 21 (class A) or 7 (class C) amino acid residues between the A and B parts of the HR-A/B motif, class A and class C Hsfs have longer HR-A/B regions than class B Hsfs, which are distinguished from classes A and C by the presence of a heptad repeat pattern instead of an insertion (Figure 5).

**Table 3:** **Functional domains and motifs of soybean Hsfs**

Gene	Subgroup	DBD	HR-A/B	NLS	NES	AHA motif
*GmHsf-24*	A1	11-104	131-182	(216) KKRR	(257) LQILQI	(399) DEFWELLL
*GmHsf-02*	A1	17-110	139-190	(224) KKRR		(407) DEILQTSV
*GmHsf-04*	A1	11-114	131-182	(216) KKRR		(399) DEFWELFL
*GmHsf-17*	A1	17-120	149-200	(234) KKRR		(446) DEILQTSV
*GmHsf-33*	A1	27-120	148-200	(233) KKRR		(456) DDILRTPV
*GmHsf-08*	A2	40-133	158-210	(240) RKRR		(312) DSVWEDLLN
*GmHsf-30*	A2	40-133	156-208	(240) RKRR		(305) DTILEDFLN
*GmHsf-34*	A2	39-132	153-204	(240) RKRR		(293) DSILEDFLN
*GmHsf-07*	A3	36-129	148-195	(233) RRRFIK	(156) LESLRKERSVL	(374) KRNTNFDVSG
*GmHsf-20*	A3	44-137	159-190	(243) VRKN		(353) IWDSGLNVSG
*GmHsf-28*	A3	1-86	105-151	(191) VRKFVK		(346) IWDSGLNVSG
*GmHsf-11*	A4	1-83	100-159	(182) DRKRR		(318) DVFWEQFLTE
*GmHsf-13*	A4	10-103	121-177	(202) DRKRR		(338) DVFWEQFLTE
*GmHsf-29*	A4	11-104	121-190	(205) DRKRR		(341) DIFWERFLTE
*GmHsf-31*	A4	11-104	122-180	(205) DRKRR		() DIFWERFLTE
*GmHsf-09*	A5	12-105	122-149	(204) YKKRR	(347) LTL	(427) DVFWEQFLTE
*GmHsf-15*	A5	10-103	119-153	(202) YKKRR	(345) LTL	(425) DVFWEQFLTE
*GmHsf-21*	A6	38-131	153-194	(222) KSK7KKRR	(160) HDKLVL	(297) DEEFWEELLFSE
*GmHsf-23*	A6	123-216	243-295	(316) WRK7NKKRR	(380) LDLALNL	(414) DEVFWQDLLNE
			384-416			
*GmHsf-22*	A6	21-114	140-197	(210) WRK7KR		(282) EEVLWEELLNE
*GmHsf-37*	A6	16-109	135-188	(208) WRK7KKRRR		(303) DEVFWQDLLNE
			278-300			
*GmHsf-10*	A8	7-100	132-170		(281) LSPLEN	
*GmHsf-14*	A8	13-106	138-181		(287) LSPLEN	
*GmHsf-01*	B1	6-99	155-188	(245) KRGR		
*GmHsf-12*	B1	8-137	179-216	(303) RKRGR		
*GmHsf-25*	B1	7-100	157-190	(248) KRGR		
*GmHsf-35*	B1	8-101	144-176	(262) RKRGR	(162) GELAL	
*GmHsf-03*	B2	4-97	162-197	(273) LKRCR	(174) LRKENMQL	
*GmHsf-18*	B2	30-123	170-200	(261) AKRAR	(186) LTKELAEMRSL	
*GmHsf-19*	B2	20-113	164-194	(237) TKRAR	(180) LTKELEEMRS	
*GmHsf-26*	B2	21-114	180-213	(294) LKRCR	(197) IQL	
*GmHsf-32*	B2	29-122	171-201	(278) AKRAR	(187) LTKELAEMRSL	
*GmHsf-38*	B2	20-113	169-195	(242) KKRAR		
*GmHsf-06*	B3	17-110	146-185	(206) QGER		
*GmHsf-36*	B3	17-112	148-184	(208) QGGR		
*GmHsf-05*	B4	31-129	150-190			
*GmHsf-27*	B4	34-132	155-194		(178) LELQM	
*GmHsf-16*	C1	13-106	131-170	(199) KKRR	(142) LKEEQKAL

Depending on the balance of nuclear import and export, the intracellular distribution of Hsfs changes dynamically between nucleus and cytoplasm [10, 25]. A hydrophobic, frequently leucine-rich NES at the C-terminal of many Hsfs is required for receptor-mediated nuclear export in a complex with the NES receptor. Together with the adjacent AHA motifs, NES serves as part of a type-specific signature region in the C-terminal of class A Hsfs in plants [26]. AHA motifs exist only in class A Hsfs (Table 3). It was noted that the α2-α3 sequence in DBD of GmHsf-12 was unique (Figure 4). An NLS was not detected in GmHsf-10, GmHsf-14, GmHsf-05, and GmHsf-27; NES was located in HR-A/B in 9 Hsf proteins (GmHsf-03, GmHsf-07, GmHsf-16, GmHsf-18, GmHsf-19, GmHsf-21, GmHsf-26, GmHsf-32, and GmHsf-35); and two HR-A/B regions were found in GmHsf-23 (Table 3).

### Expression patterns of soybean Hsf genes

**Figure 6:**
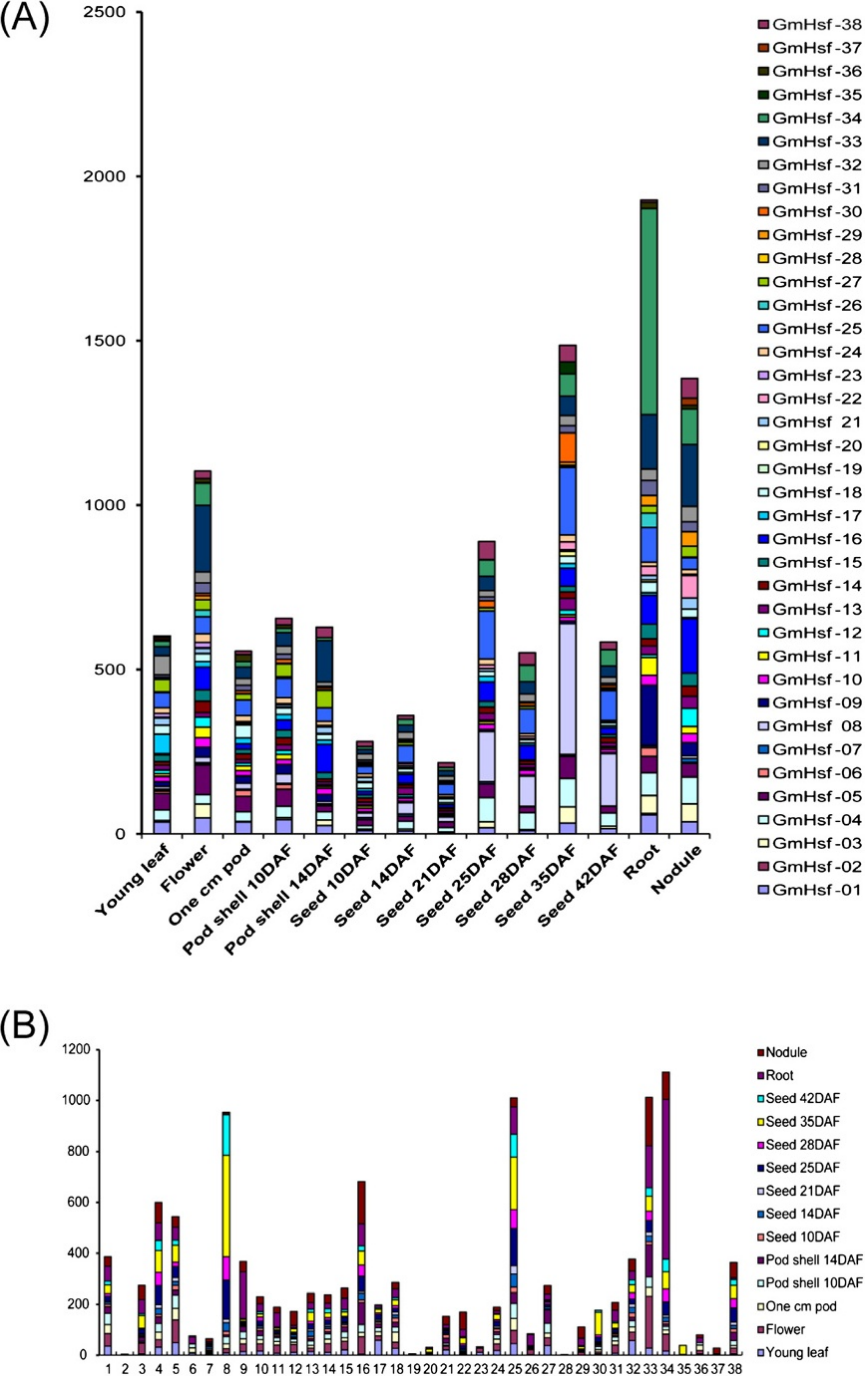
**Expressions of soybean Hsf genes. (A)** Expression levels of all soybean Hsf family genes in tissues and developmental stages. **(B)** Expression levels of each soybean Hsf family genes. The normalized expression data of the soybean Hsf genes were collected from the SoyBase (http://www.soybase.org/). The expression unit (vertical coordinates) is transcripts per million (TPM).

To examine expression patterns in different soybean tissues and organs, an expression pattern map of soybean Hsf genes based on the gene-chip data downloaded from the soybean genome database was drawn (Figure 6 and Additional file 1: Table S1). The data analysis revealed that soybean Hsf genes were expressed in 14 tissues and organs and at different developmental stages. Moreover, soybean Hsf genes were expressed at the highest level in roots and at the lowest level in seeds after 21 days of development (Figure 6A).

Three soybean Hsf genes showed tissue-specific expression patterns. For example, *GmHsf-02* was expressed in roots; *GmHsf-28* in roots and seeds after 14 days of development; and *GmHsf-37* in young leaves and root nodules. *GmHsf-02*, *GmHsf-19*, and *GmHsf-28* expressed at a low level, whereas *GmHsf-08*, *GmHsf-25*, *GmHsf-33*, and *GmHsf-34* at an extremely high level. Expression levels were disparate in different soybean Hsf subclasses. Compared with others, the expression levels for subclass A3 were lower. Even in the same subclass, expression levels were varied. For example, *GmHsf-17* transcripts reached maximum levels in young leaves, whereas *GmHsf-33* reached maximum levels in flowers and pod shells at 14 DAF, and also in nodules. In addition, data from the tissue expression chip revealed differences in expression between 15 pairs of paralogous genes. For example, although *GmHsf-20* and *GmHsf-28* were expressed at relatively low levels, *GmHsf-20* was expressed in 8 tissues and organs, and *GmHsf-28* was expressed only in seeds 14 DAF and in roots at a very low level; Although *GmHsf-23* was expressed much like *GmHsf-37* in quantity, *GmHsf-37* was expressed only in young leaves and nodules, whereas *GmHsf-23* was also expressed in flowers and seeds at 35 DAF.

### qRT-PCR analyses of soybean Hsf genes

**Figure 7:**
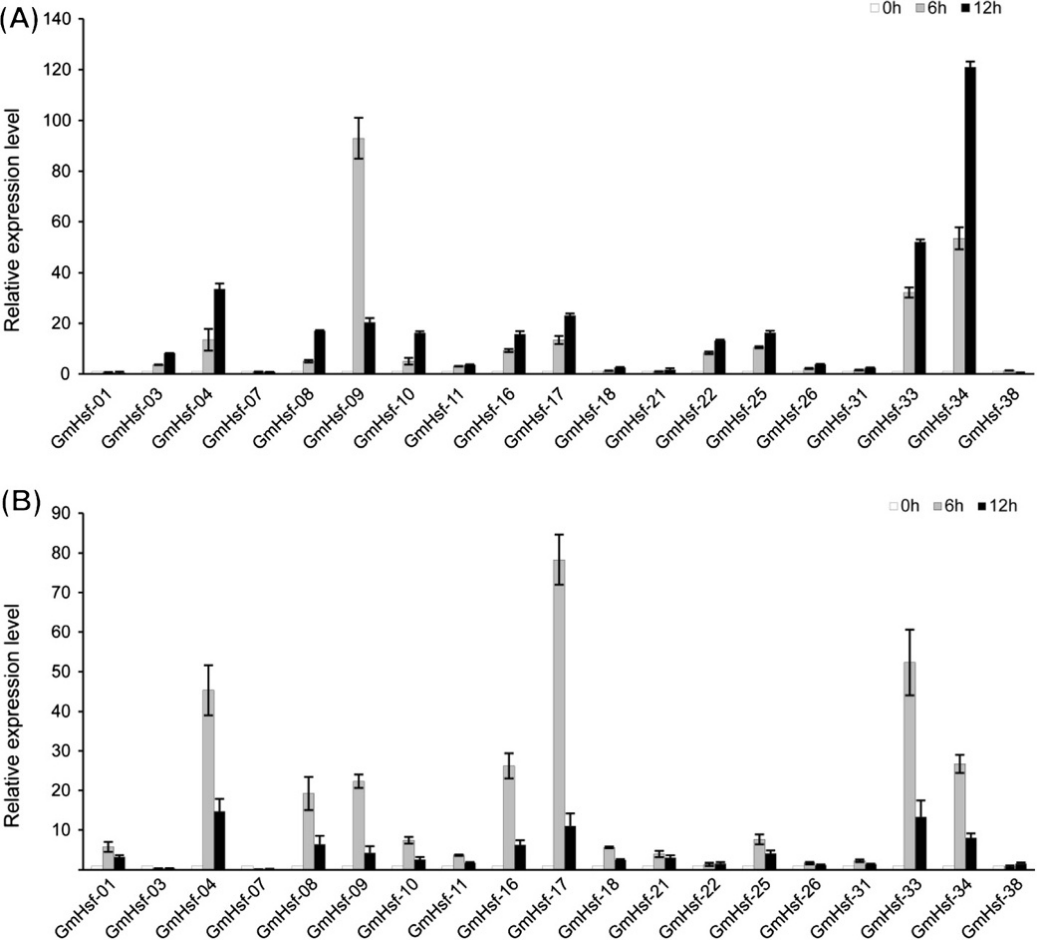
**Relative expressions of soybean Hsf genes under drought and heat conditions.** Analyses were carried out by qRT-PCR under drought **(A)** and heat stress **(B)** treatment. qRT-PCR data were normalized using soybean *Actin* (U60506) gene and shown relative to 0 h. X-axes showed soybean Hsf genes and y-axes are scales of relative expression level (error bars indicate SD).

Nineteen soybean Hsf genes which expressed highly in different tissues were selected to further confirm their responses to drought stress and heat stress. qRT-PCR was carried out using soybean plants exposed to drought (0, 6, and 12 h) and high temperature (0, 6, and 12 h). These genes expressed diversely under both stresses (Figure 7A, B); 14 genes were up-regulated (>2-fold) by drought stress, and 13 were up-regulated by heat stress. Notably, 10 soybean Hsf genes (*GmHsf-04*, *GmHsf-08*, *GmHsf-09*, *GmHsf-10*, *GmHsf-11*, *GmHsf-16*, *GmHsf-17*, *GmHsf-25*, *GmHsf-33*, and *GmHsf-34*) showed up-regulation under both drought and heat stress conditions. Two soybean Hsf genes (*GmHsf-03* and *GmHsf-07*) were greatly down-regulated (<0.5-fold) during the heat stress treatment. Moreover, the transcript level of *GmHsf-38*, was unchanged by either stress.

### GmHsf-04, GmHsf-33, and GmHsf-34 were localized in the nucleus

**Figure 8:**
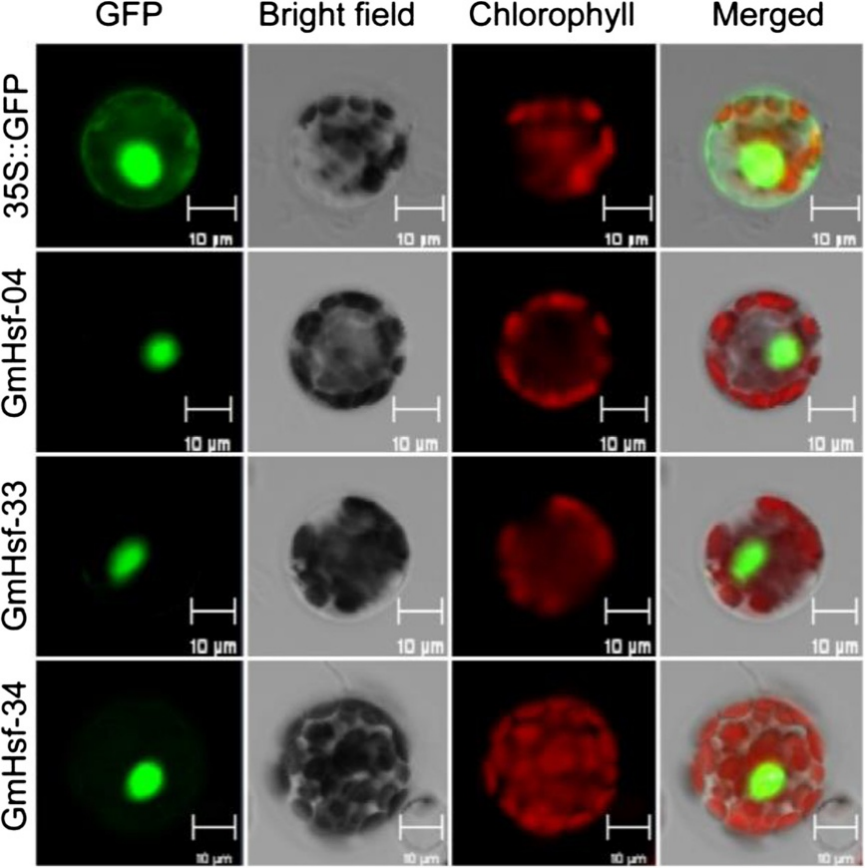
**Subcellular localization of GmHsf proteins.** The 35S::GmHsf-04-GFP, 35S::GmHsf-33-GFP, 35S::GmHsf-34-GFP and 35S::GFP control vectors were transiently expressed in *Arabidopsis* protoplasts. Results were visualized by a confocal microscopy 16 h after transformation. Bars =10 μm.

Three genes (*GmHsf-04*, *GmHsf-33*, and *GmHsf-34*) up-regulated strongly by both heat and drought were selected for subcellular localization. Expression vectors with green fluorescent protein (GFP) tags were constructed for subcellular localization analysis. The coding regions of *GmHsf-04*, *GmHsf-33*, and *GmHsf-34* were amplified from the soybean cDNA by PCR with specific primers and fused to the N-terminal of GFP under control of the CaMV 35S promoter. Subcellular localization of GFP expression in mesophyll cell protoplasts of *Arabidopsis* was monitored by confocal microscopy 16 h after transformation mediated by PEG; 35S::GFP vector was transformed as the control. As shown in Figure 8, control hGFP was uniformly distributed throughout the mesophyll cell protoplast, whereas GmHsf-04, GmHsf-33, and GmHsf-34 fusion proteins were exclusively localized in the nucleus. These results suggest that GmHsf-04, GmHsf-33, and GmHsf-34 are nuclear proteins, possibly serving as transcription factors.

### Overexpression of *GmHsf-34*improved tolerance to drought and heat stresses in *Arabidopsis*

**Figure 9:**
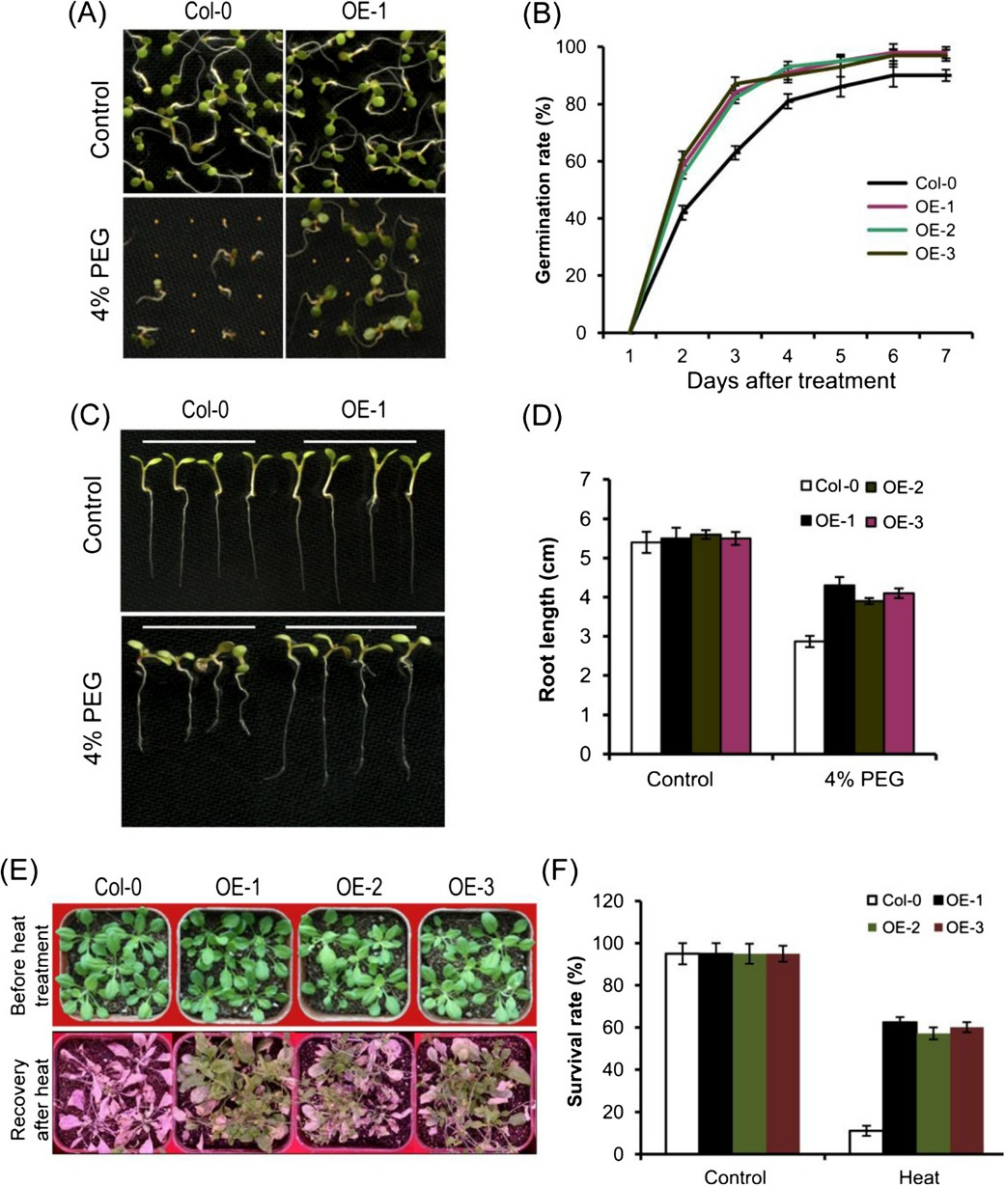
**Responses of** ***GmHsf-34*** **overexpressing** ***Arabdopsis*** **to drought and heat stresses** ***.*** **(A)** Seed germinations of wild-type (Col-0) and *GmHsf-34* overexpressing *Arabdopsis* seeds. Seeds from three independent transgenetic lines of *GmHsf-34* were grown on ½ MS medium with or without 4% PEG. Wild-type seeds were grown in the same condition as a control. Representative images of wild-type and one line (OE-1) of the transgenic plants were taken after treated by 4% PEG for 4 days. **(B)** Germination rates of wild-type and *GmHsf-34* overexpressing *Arabdopsis* seeds. Germination rates were determined daily after 2 days stratification. Data represent means ± SD (n =90). **(C)** and **(D)** Root lengths of wild-type and *GmHsf-34* overexpressing plants. Root length was measured by the ImageJ 2X software. Data represent means ± SD (n =90). **(E)** Representative images of wild-type and transgenic plants after treated under high temperature conditions. Four-week-old wild-type and *GmHsf-34* overexpressing seedlings (OE-1, OE-2, and OE-3) were exposed to 42°C for 6 hours heat stress, and then transferred to normal condition. After one week, the photographs were taken. Three independent experiments were conducted. **(F)** Survival rates of wild-type and *GmHsf-34* overexpressing plants after heat stress. One week after heat treated, survival rate of wild-type and transgenetic plants. Data represent means ± SD (n =90).

According to the expression analysis, *GmHsf-34* was strongly induced by drought and heat stresses. To confirm the functions of *GmHsf-34* in abiotic stress response, three lines of *Arabidopsis* overexpressing *GmHsf-34* were tested under drought and high temperature conditions, respectively. Seed germination and root growth of transgenic *Arabidopsi*s were tested in the presence of 4% PEG (Figure 9A to D). Under standard culture conditions, no significant differences in germination rate or morphology between transgenic and wild-type (Col-0) plants were observed. However, germination percentage of transgenic plants was enhanced by nearly 15% compared to wild-type after 2-3 days (Figure 9A and B). In the presence of 4% PEG, roots of transgenic lines were longer than those of wild-type plants (Figure 9C and D), showing that overexpression of *GmHsf-34* improved tolerance to the imposed drought treatment in *Arabidopsis*. After heat stress treatment, survival rates of *Arabidopsis* seedlings overexpressing *GmHsf-34* and wild-type were recorded (Figure 9E and F). The survival rate of wild-type *Arabidopsis* seedlings was about 11.5%, whereas that of *Arabidopsis* seedlings overexpressing *GmHsf-34* was improved to 58.5-62.5%. Obviously, the transgenic seedlings displayed higher tolerance to high temperature compared to wild-type plants.

## Discussion

In previous work, 59 soybean Hsfs presented from the soybean Transcription Factor Database website (http://soybeantfdb.psc.riken.jp/) were reported [27]. In this study, although a comprehensive set of 58 possible soybean Hsfs were obtained after scanning the current version of the soybean genome (JGI Glyma1.0 annotation), 38 nonredundant soybean Hsfs were finally identified and characterized. Comparison with the locus numbers indicated that soybean Hsfs identified in our study were completely included in reported 59 soybean Hsfs. The widely accepted model of Hsfs defines the necessity of Hsf-type DBD and OD characterized by coiled-coil structure [19]. Briefly, DBD ensures the Hsfs combination with HSEs, and coiled-coil domain is indispensable for trimerization leading to Hsfs activity. Consequently, we surveyed and discarded extra 21 similar Hsfs due to the absence of Hsf-type DBD domains and/or coiled-coil structures.

The monocots rice and maize contain the same number of *Hsf* genes (25), whereas the numbers in dicots soybean (38) and *Arabidopsis* (21) are quite different. This probably results from the double duplications of genome in soybean [28] but only a single replication in *Arabidopsis* [29] during evolution. The cluster analysis indicated that Hsfs in the same subclasses in *Arabidopsis* and soybean, or maize and rice, belong to the same branch in accord with the evolutionary relationships of *Arabidopsis* and soybean being dicots and maize and rice being monocots. Several genes are unique to monocots or dicots. For example, the subclasses A8 and B3 are restricted to dicots, and A9 and C2 are characteristic of monocots, suggesting the evolution of these subclasses followed the divergence of monocots and dicots. In addition, the subclass A7 is absent in soybean, presumably was lost in the processes of gene recombination, mutation, or redundancy.

In recent years, research on the role of introns has made significant progress. Studies in mammals, nematodes, insects, fungi, and plants suggest that the introns not only regulate the gene expression, but also participate in the gene evolution [30]. Analysis of gene structures revealed that soybean Hsf genes contain a single intron except *GmHsf-12* containing two introns. Combined with analysis of the gene chip expression results (Figure 6), the soybean Hsf gene *GmHsf-12* was not expressed at lower levels than others under the normal conditions. Seemingly, an intron does not affect gene expressions. Combined with the analysis to phylogenetic evolution (Figure 1), the number and location of *Hsf* intron in the same subclass are conserved (Figure 3). For example, *GmHsf-30* and *GmHsf-34* in subclass A2 respectively at 307 bp to 2110 bp and 304 bp to 2113 bp section contains an intron, which indicated that introns could be a reference to the evolution of plant genes.

Transcriptional activity of class A Hsfs is normally mediated by the AHA motif in the C-terminal region. However, the AHA motif is absent in GmHsf-10 or GmHsf-14 in the A3 subclass in soybean (Table 3). It was proposed that proteins without an AHA motif were activated through formation hetero-polymers with other class A Hsfs [17]. Unlike class A Hsfs, most of the class B and C Hsfs do not have the transcription activation ability, since their CTDs lack detectable the AHA motifs. Instead, the class B of Hsfs is characterized with a tetrapeptide-LFGV- in the C-terminal region, which is assumed to function as a repressor motif in the transcription machinery. The previous research showed that several other transcription factors functioning as the repressors also contain a conservative tetrapeptide-LFGV-motif, such as ABI3/VP1, AP2/ERF, MYB and GRAS, although their mechanisms of the action remain unclear [31, 32].

The signal transduction pathways are complicated networks where components work together to control the plant physiological and biochemical process. AREB1 (ABRE-BINDING PROTEIN 1), AREB2, and ABF3, members of class A bZIP transcription co-factors of the ABRE elements, regulate the response to osmotic stress through combining with the element in the *DREB2A* promoter region [33]. The analysis of *cis*-acting elements in their promoter regions revealed that the soybean Hsf genes contain the MYB/MYC elements and some contain the ABRE, DRE, and/or LTRE elements, demonstrating that the soybean Hsfs play significant roles in the regulation of stress responses (Table 2). The MYB elements basically participate in the drought, low temperature, salt, ABA, and GA stress responses [34, 35] and the MYC elements participate in the drought, salt, and ABA stress responses. We show that the *Arabidopsis*, rice, and maize Hsf gene promoters contain the MYB/MYC elements (Table 2) and we concluded that Hsfs are involved in the responses to drought, salt, and ABA in plants. However, the expression results from the gene chips did not agree perfectly with the conclusion. For instance, *GmHsf-25* contained 15 *cis*-acting elements and its expression value was up to 1090, whereas the value for *GmHsf-10* carrying 85 *cis*-acting elements was only 228 (Table 2 and Additional file 1: Table S1). One explanation may be that, several elements lost their activities or performed in a negative way.

In consideration of putative similar functions of Hsf genes in the same subclass, 19 genes containing all subclass were selected to investigate responses to drought and heat stresses using qRT-PCR. The results showed that the soybean Hsf genes were differently expressed under the drought stress and heat stress conditions. We detected that three soybean Hsfs (*GmHsf-04*, *GmHsf-17*, and *GmHsf-33*) belonged to the subclass A1 were expressed at significantly high levels under the drought stress and heat stress (Figure 7). Moreover, *GmHsf-08* and *GmHsf-34* (subclass A2), *GmHsf-11* (subclass A4), *GmHsf-09* (subclass A5), *GmHsf-10* (subclass A8), *GmHsf-25* (subclass B1), and *GmHsf-16* (subclass C1) were up-regulated under both drought stress and heat stress conditions. It was reported that tomato HsfA1a and *Arabidopsis* HsfA2 function as master regulators for acquired thermo-tolerance [36, 37], and tomato HsfB1 was a co-regulator with HsfA1a [24]. These results indicate that A1, A2, and several other subclass members may be involved in the drought stress and heat stress responses in plants. According to the soybean gene chip data, *GmHsf-02*, *GmHsf-07*, *GmHsf-19*, *GmHsf-20*, *GmHsf-28*, *GmHsf-35*, and *GmHsf-37* were expressed at very low levels. Among them, *GmHsf-07*, *GmHsf-20*, and *GmHsf-28* belong to subclass A3. It is likely that the functions of these soybean Hsf genes, especially subclass A3, are not related to drought or heat stress responses. Most of the soybean Hsf genes expressed in roots were regulated by drought while those expressed in young leaves were regulated by heat. This is consistent with the fact that the root is the first organ sensing drought stress whereas leaf is first to experience heat stress.

In the former publication [27], expression analyses of 5 soybean Hsf genes (*GmHsf12*, *GmHsf28*, *GmHsf34*, *GmHsf35*, and *GmHsf47*) were performed under heat, low-temperature, NaCl, and drought stresses, respectively. These 5 genes were named in our study as *GmHsf-08*, *GmHsf-21*, *GmHsf-26*, *GmHsf-25*, and *GmHsf-32* respectively. We founded that *GmHsf-08*, *GmHsf-21*, and *GmHsf-25* showed markedly up-regulation by heat, which was consistent with the former works, while *GmHsf-26* showed no detectable alteration. Under drought condition, *GmHsf-08*, *GmHsf-25*, and *GmHsf-*26 showed to be up-regulated strongly while *GmHsf-21* expression was not influenced. *GmHsf-32* expressions under abotic conditions were not surveyed in our study. *GmHsf-34* was strongly induced by drought and heat stresses and its overexpression improved survival rate and/or root development in *Arabidopsis* under simulated drought and heat conditions (Figure 9). Similarly, overexpression of *Arabidopsis AtHsfA2*, one of the most strongly induced genes, lead to enhanced tolerance to heat stress [38]. Given the close phylogenetic relationship of *GmHsf-34* with *AtHsfA2*, it is speculated that GmHsf-34 functions as a typical transcription factor due to the existence of Hsf-type DBD, OD, NES, NLS, and AHA motifs, and participates in heat and drought responses.

## Conclusions

Thirty eight soybean Hsf genes were initially identified and classified after scanning for the soybean genome data base. Their locations and duplications, intron-exon structures, DBD structures and HR-A/B, distribution of *cis*-acting elements in the soybean Hsf promoters, and the expression patterns were determined. Based on the expression analysis, we inferred that soybean Hsf subclasses A1 and A2 may be the primary regulators of the heat stress response in soybean. *GmHsf-34*, a member of subclass A2, played an important role in the response to the drought and heat stress treatments imposed in this study.

## Methods

### Database searches for Hsf genes in soybean, *Arabidopsis*, and rice genomes

The whole genome data and the repeat information of soybean and maize were obtained from JGI Glyma1.0 annotation [39]. The gene sequences and protein sequences of *Arabidopsis* and rice Hsfs were acquired from TAIR [40], and TIGR [41], respectively. The gene chip data of soybean were derived from SoyBase [42].

### Identification and physical locations of soybean Hsfs

To gather the probable candidate soybean Hsf amino acid sequences, the Hsf-type DBD domain (Pfam: PF00447) was submitted as a query in a BLASTP (P = 0.001) search of the soybean genome data base. A total of 38 soybean Hsfs were obtained after manually filtering out repeated sequences, and sequences without integrated Hsf-type DBD domains or classic coilled-coil structures by SMART [43]. All non-redundant Hsfs were mapped on the 20 soybean chromosomes on the basis of the information in the soybean database using MapDraw software [44]. The paralogous genes are identified and connected by lines according to Lin’s method [18].

### Genetic structure and *cis*-acting elements

An exon-intron substructure map was produced by Tools Online GSDS [45], and Promoter 2.0 [46] was applied to predict the soybean Hsf promoters. *Cis*-acting elements were analyzed by referring to the plant *cis*-acting element database PLACE26.0 [47].

### Domain prediction and phylogenetic relationships

Clustal X 2.0 [48] was applied in protein sequence comparison analyses of Hsfs in *Arabidopsis*, rice, and soybean. Database tools Pfam [49], SMART, PredictNLS [50] and NetNES [51] were consulted to analyze their typical functional structure domains. A phylogenetic tree was constructed using the adjacent method by MEGA5.0 [52] with a 1000 bootstrap value.

### Expression patterns

An analysis was conducted using the soybean gene chip expression data, the analysis was carried out which included the 38 soybean Hsfs in the different tissues and development stages, and also the diversity of different genes, subclasses, organs, tissues, and development stages.

### Plant materials and stress treatments

The soybean seeds were germinated in the vermiculite in a light chamber at 25°C for 14 days. The soybean seedlings were removed and exposed to a heat stress temperature (42°C) for 0, 6, and 12 h, after which they were sampled for RNA extraction. For the drought stress, soybean seedlings were removed from the soil, and dehydrated for 0, 6, and 12 h before being sampled and frozen in liquid nitrogen and stored at -80°C.

### RT-PCR and qRT-PCR

The total RNA was isolated from the whole plants using an RNeasy Plant Mini Kit (Qiagen) according to the manufacture’s handbook. The cDNA synthesis and reverse transcription-PCR (RT-PCR) were conducted as previously described [53]. Quantitative real-time PCR (qRT-PCR) for examination of the soybean Hsfs were performed with the SYBR Premix ExTaqTM kit (TaKaRa) and an ABI 7300 according to the manufacturer’s protocols (Applied Biosystem). The expression patterns were analyzed with an ABI Prism 7300 sequence detection system (Applied Biosystems) as previously described [54]. The soybean Hsf genes primers for qRT-PCR were designed using the Primer Premier 5.0 software avoiding the Hsfs conservative domain and soybean *Actin* (U60506) was used as an internal control for normalization of the template cDNA.

### Subcellular localization in *Arabidopsis*protoplasts

The expression vectors with green fluorescent protein (GFP) tags were constructed for the subcellular localization analysis as described previously [55]. The coding regions of *GmHsf-04*, *GmHsf-33*, and *GmHsf-34* were amplified by PCR using the specific primers and fused to the N-terminal of GFP under control of the CaMV35S promoter. The subcellular localization of the GFP expression in the *Arabidopsis* protoplasts was monitored by confocal microscopy 16 h after polyethylene glycol mediated transformation as described [56].

### Tolerance assays under stress conditions

The *GmHsf-34* gene, which is induced by the drought and heat stresses, was selected to confirm gene functions. Expression vector pBI121::GmHsf-34 containing *GmHsf-34* under control of the CaMV35S promoter was built. Three *Arabidopsis* lines overexpressing *GmHsf-34* were obtained after the transformation mediated by agrobacterium (*Agrobacterium tumefaciens*). For the germination assays, the seeds of Col-0 and transgenic plants were placed on ½ MS medium containing no or 4% (w/v) PEG for 7 days. For the root growth assays, 4-day-old seedlings grown on ½ MS medium were transferred to ½ MS medium containing no or 4% (w/v) PEG for 4 days after which the root lengths were measured by a root system scanner. For the heat stress tolerance assays, the 21-day-old seedlings grown in the soil transferred from ½ MS medium were treated at 42°C for 6 h and then grown under the normal condition for several days.

## Electronic supplementary material

Additional file 1: Table S1: Normalized digital gene expression counts of the uniquely mappable reads of soybean Hsf genes. For informations collection of gene expressions, ID numbers of soybean Hsf genes were submitted into Soybase [http://soybase.org/soyseq/]. (DOC 98 KB)

## Abbreviations

ABRE: ABA-responsive element
AREB: ABRE binding protein
DBD: DNA-binding domain
DRE: Dehydration-responsive element
DREB: DRE binding protein
GFP: Green fluorescent protein
HSE: Heat stress element
Hsf: Heat shock transcription factor
Hsp: Heat shock proteins
LTRE: Low-temperature responsive element
NES: Nuclear export signal
NLS: A nuclear localization signal
OD: Oligomerization domain.
