# Supplementary material for: Genome-wide analysis of the Hsf family in soybean and functional identification of GmHsf-34 involvement in drought and heat stresses
Source: BMC Genomics. 2014 Nov 21;15(1):1009. doi: 10.1186/1471-2164-15-1009 (PMC4253008; doi:10.1186/1471-2164-15-1009)
Supplement: Supplementary file 1 — Additional file 1: Table S1: Normalized digital gene expression counts of the uniquely mappable reads of soybean Hsf genes. For informations collection of gene expressions, ID numbers of soybean Hsf genes were submitted into Soybase [http://soybase.org/soyseq/]. (DOC 98 KB) [file 12864_2014_6707_MOESM1_ESM.doc]

**Additional file 1: Table S1.** Digital expression analysis of HSF genes

| Gene names | Young leaf | Flower | One cm pod | Pod shell 10DAF | Pod shell 14DAF | Seed 10DAF | Seed 14DAF | Seed 21DAF | Seed 25DAF | Seed 28DAF | Seed 35DAF | Seed 42DAF | Root | Nodule |
| --- | --- | --- | --- | --- | --- | --- | --- | --- | --- | --- | --- | --- | --- | --- |
| *GmHsf-01* | 36 | 49 | 36 | 44 | 26 | 10 | 9 | 3 | 19 | 10 | 33 | 16 | 58 | 37 |
| *GmHsf-02* | 0 | 0 | 0 | 0 | 0 | 0 | 0 | 0 | 0 | 0 | 0 | 0 | 4 | 0 |
| *GmHsf-03* | 5 | 42 | 2 | 6 | 16 | 5 | 6 | 3 | 18 | 3 | 50 | 8 | 55 | 55 |
| *GmHsf-04* | 32 | 29 | 30 | 34 | 26 | 10 | 24 | 14 | 74 | 52 | 86 | 40 | 68 | 81 |
| *GmHsf-05* | 50 | 89 | 46 | 52 | 18 | 19 | 15 | 16 | 42 | 18 | 67 | 20 | 51 | 41 |
| *GmHsf-06* | 8 | 2 | 19 | 15 | 1 | 0 | 0 | 0 | 0 | 0 | 1 | 0 | 26 | 3 |
| *GmHsf-07* | 5 | 5 | 3 | 3 | 5 | 6 | 7 | 1 | 6 | 1 | 4 | 1 | 6 | 12 |
| *GmHsf-08* | 9 | 17 | 19 | 28 | 7 | 14 | 34 | 14 | 152 | 92 | 399 | 159 | 1 | 8 |
| *GmHsf-09* | 14 | 30 | 21 | 28 | 21 | 4 | 5 | 2 | 6 | 3 | 7 | 3 | 183 | 40 |
| *GmHsf-10* | 16 | 29 | 16 | 16 | 19 | 10 | 9 | 4 | 16 | 15 | 13 | 9 | 30 | 28 |
| *GmHsf-11* | 8 | 32 | 14 | 15 | 6 | 7 | 3 | 3 | 8 | 3 | 7 | 6 | 55 | 22 |
| *GmHsf-12* | 11 | 31 | 9 | 13 | 5 | 3 | 8 | 2 | 5 | 4 | 14 | 3 | 9 | 55 |
| *GmHsf-13* | 14 | 14 | 11 | 17 | 9 | 9 | 21 | 8 | 21 | 10 | 35 | 12 | 26 | 36 |
| *GmHsf-14* | 11 | 35 | 17 | 21 | 9 | 12 | 8 | 7 | 18 | 10 | 19 | 16 | 22 | 32 |
| *GmHsf-15* | 21 | 34 | 14 | 24 | 18 | 10 | 6 | 4 | 18 | 4 | 18 | 9 | 44 | 39 |
| *GmHsf-16* | 4 | 69 | 17 | 31 | 86 | 11 | 25 | 8 | 59 | 44 | 55 | 20 | 87 | 166 |
| *GmHsf-17* | 59 | 17 | 17 | 16 | 13 | 8 | 6 | 6 | 18 | 7 | 14 | 6 | 8 | 3 |
| *GmHsf-18* | 27 | 24 | 40 | 20 | 18 | 18 | 12 | 12 | 14 | 10 | 23 | 9 | 32 | 26 |
| *GmHsf-19* | 0 | 0 | 1 | 0 | 3 | 1 | 0 | 0 | 0 | 0 | 0 | 0 | 1 | 0 |
| *GmHsf-20* | 1 | 1 | 2 | 2 | 0 | 2 | 3 | 0 | 0 | 0 | 14 | 0 | 6 | 0 |
| *GmHsf-21* | 21 | 15 | 4 | 8 | 19 | 13 | 6 | 2 | 9 | 3 | 5 | 1 | 14 | 33 |
| *GmHsf-22* | 3 | 2 | 3 | 3 | 5 | 1 | 6 | 3 | 11 | 8 | 23 | 5 | 28 | 69 |
| *GmHsf-23* | 11 | 17 | 0 | 0 | 0 | 0 | 0 | 0 | 0 | 0 | 1 | 0 | 0 | 4 |
| *GmHsf-24* | 18 | 26 | 19 | 18 | 12 | 10 | 3 | 8 | 18 | 8 | 21 | 2 | 12 | 14 |
| *GmHsf-25* | 46 | 52 | 47 | 59 | 41 | 22 | 52 | 32 | 146 | 75 | 206 | 91 | 106 | 35 |
| *GmHsf-26* | 2 | 20 | 1 | 5 | 1 | 2 | 1 | 1 | 1 | 0 | 2 | 1 | 44 | 3 |
| *GmHsf-27* | 38 | 31 | 18 | 39 | 53 | 3 | 9 | 1 | 9 | 8 | 5 | 4 | 22 | 33 |
| *GmHsf-28* | 0 | 0 | 0 | 0 | 0 | 0 | 1 | 0 | 0 | 0 | 0 | 0 | 1 | 0 |
| *GmHsf-29* | 1 | 11 | 1 | 2 | 1 | 3 | 4 | 0 | 1 | 1 | 9 | 3 | 30 | 44 |
| *GmHsf-30* | 5 | 8 | 9 | 12 | 7 | 5 | 2 | 4 | 19 | 9 | 89 | 8 | 1 | 0 |
| *GmHsf-31* | 8 | 32 | 16 | 16 | 4 | 7 | 3 | 4 | 12 | 4 | 21 | 6 | 45 | 30 |
| *GmHsf-32* | 58 | 33 | 21 | 24 | 13 | 18 | 21 | 14 | 19 | 24 | 31 | 19 | 35 | 47 |
| *GmHsf-33* | 28 | 204 | 34 | 41 | 126 | 13 | 22 | 16 | 44 | 37 | 59 | 34 | 165 | 189 |
| *GmHsf-34* | 16 | 67 | 17 | 14 | 9 | 9 | 17 | 10 | 50 | 50 | 68 | 50 | 627 | 107 |
| *GmHsf-35* | 0 | 1 | 0 | 0 | 0 | 0 | 0 | 0 | 1 | 1 | 36 | 0 | 0 | 0 |
| *GmHsf-36* | 5 | 13 | 22 | 10 | 0 | 0 | 0 | 0 | 0 | 0 | 0 | 0 | 19 | 11 |
| *GmHsf-37* | 6 | 0 | 0 | 0 | 0 | 0 | 0 | 0 | 0 | 0 | 0 | 0 | 0 | 22 |
| *GmHsf-38* | 5 | 23 | 10 | 20 | 32 | 15 | 12 | 14 | 55 | 37 | 51 | 23 | 7 | 60 |
